# Genetic diversities and drug resistance in *Mycobacterium bovis* isolates from zoonotic tuberculosis using whole genome sequencing

**DOI:** 10.1186/s12864-024-10909-8

**Published:** 2024-11-01

**Authors:** Noha Salah Soliman, May Sherif Soliman, Sahar Mohammed Khairat, Maha Ali Gad, Sherine Shawky, Amani Ali Elkholy

**Affiliations:** 1 Clinical and Chemical Pathology, Faculty of Medicine, Cairo University, Cairo, Egypt; 2 Microbiology Department, Medical Research Institute, Alexandria University, Alexandria, Egypt

**Keywords:** Zoonotic tuberculosis, *Mycobacterium bovis*, Whole genome sequencing, Drug resistance

## Abstract

**Background:**

Zoonotic human tuberculosis (TB) caused by *Mycobacterium bovis* (*M. bovis*) is as vital as *Mycobacterium tuberculosis*, however with scarce available information. We aimed to use whole-genome sequencing (WGS) technology to take a deep insight into the circulating genotypes of human *M. bovis* and the genomic characteristics underlying virulence and drug resistance.

**Methods:**

The study included smear positive Ziehl-Neelsen samples from patients with suspected tuberculosis. Samples were cultured on Lowenstein-Jensen media and suspected colonies of *M. bovis* were selected to undergo DNA extraction and WGS. Data was analysed using the Bacterial and Viral Bioinformatics Resource Center (BV-BRC), and online bioinformatics tools. A phylogenetic tree was constructed for our sequenced strains, in addition to a set of 59 previously sequenced *M. bovis* genomes from different hosts and countries.

**Results:**

Out of total 112 mycobacterial positive cultures, five *M. bovis* were isolated and underwent WGS. All sequenced strains belonged to *Mycobacterium tuberculosis var bovis*, spoligotype BOV_1; BOV_11. Resistance gene mutations were determined in 100% of strains to pyrazinamide (*pncA* and *rpsA)*, isoniazid (*KatG* and *ahpC*), ethambutol (*embB*,* embC*,* embR* and *ubiA*), streptomycin (*rpsl*) and fluoroquinolones (*gyrA* and *gyrB*). Rifampin (*rpoB* and *rpoC*) and delamanid (*fbiC*) resistance genes were found in 80% of strains. The major represented virulence classes were the secretion system, cell surface components and regulation system. The phylogenetic analysis revealed close genetic relatedness of three sequenced *M. bovis* strains to previous reported cow strains from Egypt and human strains from France, as well as relatedness of one *M. bovis* strain to four human Algerian strains. One sequenced strain was related to one cow strain from Egypt and a human strain from South Africa.

**Conclusions:**

All sequenced *M. bovis* isolates showed the same spoligotype, but diverse phylogeny. Resistance gene mutations were detected for anti-TB drugs including pyrazinamide, isoniazid, streptomycin, ethambutol, fluoroquinolones, cycloserine, rifampin and delamanid. The virulence profile comprised genes assigned mainly to secretion system, cell surface components and regulation system. Phylogenetic analysis revealed genetic relatedness between our isolates and previously sequenced bovine strains from Egypt as well as human strains from other nearby countries in the region.

**Supplementary Information:**

The online version contains supplementary material available at 10.1186/s12864-024-10909-8.

## Background

Tuberculosis (TB) is an ancient disease that has existed, since the beginning of the humanity. Despite global attention and efforts to control and manage tuberculosis, it remains a public health threat with devastating consequences for both humans and animals [1]. Human TB is primarily caused by *Mycobacterium tuberculosis* (*MTB*), the chief member of *Mycobacterium tuberculosis* complex (*MTB*C). However this complex comprises other mycobacterial species that have > 99% genomic identity with *MTB*, including *Mycobacterium bovis* (*M. bovis*) [2], which is the main etiological agent for bovine tuberculosis in animals [3]. Nevertheless, *M. bovis* can infect different mammalian species and, can be transmitted to humans causing zoonotic tuberculosis, which poses a global burden on public health [4]. Humans can be infected by *M. bovis*, either by inhaling aerosols that cause pulmonary tuberculosis, or by close contact with infected livestock, or by eating contaminated unpasteurized dairy products or improperly cooked food, causing extra-pulmonary tuberculosis [5, 6]. Furthermore, immunocompromised patients can often contract zoonotic tuberculosis from the *M. bovis* used in the preparation of the BCG vaccine [7]. Cases of zoonotic TB are more witnessed in countries with low socioeconomic standards, due to inevitable frequent human exposure to domestic animals, with a rate of 10% in developing countries compared to 1% in developed countries [1]. In 2016, the World Health Organization (WHO) reported human zoonotic TB infection in 147,000 people, substantially from regions in Africa and Asia [8]. However, accurate reports on the rates of human infections by *M. bovis* are yet, ill- defined and underestimated all over the world, due to the indiscernible clinical and radiological features of *MTB* and *M. bovis* infections [1, 9]. In Egypt, reports on the burden of human TB by *M. bovis* are still insufficient due to defective surveillance programs [10].

*M. bovis* shares common microbiological and biochemical traits with other mycobacterial species in the *MTB*C group, making phenotypic differentiation between them challenging, particularly by conventional culture methods [1]. For this reason, infections by *M. bovis* may be overlooked, resulting into unsuccessful diagnosis and treatment, especially with the inherent resistance of *M. bovis* to pyrazinamide and rising reports of emerging resistance to other anti-tuberculous drugs [11]. Currently available molecular tests such as multiplex PCR and hybridization methods have improved the detection and identification of *MTB*C [1]. However, these methods are still limited by their low-resolution power in discrimination between mycobacterial subspecies and the lack of information on resistance to anti-TB drugs. This is profounded by the continuous genomic evolution of *MTB*C and diversities across different populations and geographic areas [4], which implies exerting sustainable research efforts to explore emerging strains and study underlying molecular epidemiology. Recently, the advent of whole-genome sequencing has enabled full, detailed and comprehensive genomic characterization of Mycobacteria, offering a high level of confident microbial identification while determining underlying genetic markers of resistance. Moreover, genomic sequencing can re-inforce the understanding of human-animal transmission dynamics and the molecular epidemiology of evolving strains [10].

From this perspective, we aim to utilize the whole-genome sequencing (WGS) technology for entire genomic characterization of *M. bovis* clinical isolates from human tuberculosis, to explore the circulating genotypes, phylogenetic relatedness and underlying virulence and drug resistance genetic markers.

## Methods

The present study was conducted with Mycobacterial isolates collected from patients clinically and radiologically suggestive of tuberculosis in Kasr-AlAiny Cairo University Hospital from January 2020 to December 2022. The study included samples that were microscopically positive for acid-fast bacilli by Ziehl-Neelsen (ZN) stain. We handled and manipulated all samples in biosafety level-3 mycobacteriology laboratory of Kasr-Alainy Cairo University Hospital. The study obtained an ethical approval from the Research Ethics Committee of the Faculty of medicine, Cairo University, approval number: N-53-2019.

### Culture of specimens for isolation of mycobacteria

Clinical specimens were digested and decontaminated with the use N- acetyl- l- cysteine (NALC) 3% NaOH method (Becton- Dickenson, USA), then centrifuged at 3000 rpm for 20 min, for concentration following manufacturer instructions [12]. The resulting pellet was cultured on Löwenstein–Jensen (LJ) medium slants (DB Difco, New York) and the remainder of the pellets was stored at -70 C for further genomic study. Mycobacterial colonies were identified based on colony morphology and presence of positive microscopic acid-fast bacilli by ZN stain (Remel Inc., San Diego, CA) [12, 13]. *Mycobacterium bovis* was presumptively identified based on colony morphology, microscopic examination and biochemical reaction [14]. The colonies of *M. bovis* differed morphologically from *M. tuberculosis* by being dysgonic moist, smoot, flat, and friable on Lowenstein-Jensen medium, giving negative reaction for nitrate reduction and niacin accumulations, and microscopically viewed as short and straight acid-fast bacilli by ZN stain. Unlikely, *M. tuberculosis* grows as eugenic, rough, raised and wrinkled colonies positive to nitrate reduction and niacin accumulations and appears as long slender acid-fast bacilli by microscopic examination [15].

### Genomic DNA extraction and isolation

All *M. bovis* strains were subcultured on LJ media. Fresh colonies were used for genomic DNA extraction using QIAamp^®^ DNA Mini kit from Qiagen [16] and as outlined before [17, 18]. Briefly, a loopful of mycobacterial culture was re- suspended in equal volume of TE buffer placed in at 80^o^C for 20 min to kill all living organisms. Then 10 µl of 100 mg/ml lysozyme was added to each tube followed by shaking incubation at 37^o^C overnight. All subsequent steps were done according to the manufacturer protocol. Quality of the DNA was verified by fluorometer (Denovix) [16–18].

### Whole genome sequencing and bioinformatics analyses

The extracted genomic DNA was used for whole genome bacterial sequencing using Illumina MiSeq Next Generation Sequencing. A total of bacterial DNA (1ng) was used in the library preparation using Nextera XT DNA Library Preparation Kit (FC-131-1096, Illumina, San Diego, CA, USA), according to the manufacturer’s instruction. Briefly, transposons were used to fragment the DNA, subsequently adapter sequences were added onto the DNA template, products were size-selected for optimum insert length, enriched and quantified. Sequencing was carried out with the MiSeq reagent kit 600 v3 (Illumina, San Diego, CA, USA) on the Illumina MiSeq, generating an average of 301 bp paired-end reads [18, 19]. For bioinformatics analysis, FASTQ files were uploaded on the BV-BRC: Bacterial and Viral Bioinformatics Resource Center. Assembly and quality checks were done by BV-BRC tools, and services with default settings, using unicycler option used as an assembler, with default settings (a depth of 100, a minimum contig length of 200 bp and a minimum coverage of 5). After the generation of contigs, genomic annotation was obtained using bacterial comprehensive genome analysis service from the BV-BRC with default parameters applied. In addition, FASTQ files were submitted to Mykrobe [20] and TB profiler online tools [21] to determine the spoligotype and to determine the mutations conferring resistance to anti-tuberculosis drugs. Virulence factors were determined using the default webpage of the virulence factor database VFDB online tool [22]. All sequences were uploaded on the NCBI under bioproject number PRJNA1019495. *M. bovis* multi-locus sequence types (MLST) and core-genome MLST (cgMLST) were assigned by submitting the sequences to the PubMLST database [23].

### Data collection and construction of the phylogenetic tree

In addition to our *M. bovis* isolates, a total of 59 *M. tuberculosis var. bovis* genomes were used to construct the phylogenetic tree. Of these 43 genomes were obtained from the BV-BRC (Keywords: *Mycobacterium bovis* and search filtered by country to nearby country), another 16 sequences were identified in scientific articles published and covered by the NCBI. They were chosen according to the nearby Middle East countries, they were from Egypt, and from Algeria. (Supplementary material 1). Among the sequences, we selected the only *M. tuberculosis var. bovis* strain sequenced in Egypt (11 strains), isolated from dairy cattle in the Nile Delta of Egypt between 2013 and 2015. Phylogenetic tree was built and edited using bacterial genome tree service from the BV-BRC with default parameters. The Codon Tree method selects single-copy PATRIC PGFams and analyses aligned proteins and coding DNA from single-copy genes using the program RAxML. Newick files were downloaded and transferred to Itol online tool for further editing.

### Statistical analysis

Data was organized and entered in a spreadsheet of Microsoft^®^ Office Excel program. We carried out statistical calculations using the SPSS (Statistical Package for the Social Science; SPSS Inc., Chicago, IL, USA) V.15. The data was described in the form of Frequencies; numbers (N) and percentages (%).

## Results

During the study period, 112 pulmonary and extra pulmonary specimens were all positive for mycobacterial growth in culture. *M. bovis* was identified in five isolates, based on phenotypic colony morphology, microscopic examination, biochemical reactions and special growth requirements. The five *M. bovis* isolates identified as S5, S20, S23, S28 and S29 were selected for further detailed genomic characterization by whole genome sequencing. The sequenced genomic DNA of *M. bovis* produced number of nucleotides ranging from 797,243 to 2,990,556 with an approximate GC content of 65.4%. The reading of genomic sequences revealed contigs and N50 values with ranges from 137 to 288 and 26,450 to 64,728, respectively. Further data describing genome size, sequence coverage and coding sequences (CDs) are detailed in Table 1. The five *M. bovis* strains sequenced in this study had genomic accession numbers as shown in Table 2 and supplementary files 1 and 2, according to the data generated from the NCBI. All sequenced strains in our study were assigned to *Mycobacterium tuberculosis var bovis* and were recognized as spoligotypes BOV_1; BOV_11. *M. bovis* strains displayed cgSTs as illustrated in Table 2, where S5 and S28 were assigned to cgST-2291, while S23 and S29 had a shared cgST-1702.

**Table 1:** Descriptive analysis of assembled and annotated genomic sequence of *M. bovis* strains

Analyzed data	*M. bovis* strains (*n* = 5)				
	S5	S20	S23	S28	S29
Total number of reads	797,243	1,432,789	1,745,064	2,990,556	1,514,697
Nb of contigs	288	143	137	186	173
N50(bp)	26,450	64,728	64,044	46,054	49,819
Genome size in base pairs	4,167,056	4,266,839	4,228,422	4,219,738	4,202,928
No. of coding sequences	4311	4259	4205	4241	4237
rRNA	3	3	3	3	3
Repeat region	49	47	50	37	50
tRNA	44	44	44	44	44
Sequencing deph (x)	24	77	91	77	54
G + Ccontents(%)	65.37	65.49	65.47	65.38	65.35

rRNA: ribosomal ribonucleic acid; tRNA, transfer ribonucleic acid

**Table 2:** NCBI data and cgMLSTs of sequenced *M. bovis*

M. bovis strains	Biosample accession number	Genome accession number	Lineage	B. Spoligotype	MLST	cgMLST	Loci matched (%)
S5	SAMN37519983	JAVTMM000000000	*M. bovis*	BOV_1;BOV_11	ST268	cgST-2291	707/744 (95.0%)
S20	SAMN37519961	JAVTLW000000000	*M. bovis*	BOV_1;BOV_11	ST215	cgST-5953	642/744 (86.3%)
S23	SAMN37519964	JAVTLX000000000	*M. bovis*	BOV_1;BOV_11	ST268	cgST-1702	741/744 (99.6%)
S28	SAMN38024412	JAXIQB000000000	*M. bovis*	BOV_1;BOV_11	ST268	cgST-2291	733/744 (98.5%)
S29	SAMN37519969	JAVTMA000000000	*M. bovis*	BOV_1;BOV_11	ST268	cgST-1702	739/744 (99.3%)

MLST: multi-locus sequence type, cgMLST: core-genome multi-locus sequence type

### Genomic resistance profile of the sequenced *M. bovis*

As regard the genotypic resistance profile of the sequenced strains, all *M. bovis* strains (100%) harboured gene mutations conferring resistance to isoniazid, streptomycin, ethambutol, fluoroquinolones and cycloserine, while resistance gene mutation to rifampin and delamanid were found in 4/5(80%) of *M. bovis* strains. Table 3 fully describes all the identified resistant genes conferred to different anti-tuberculous drugs, and the relevant point mutations. Two of the sequenced *M. bovis* strains (S5, S20) showed pre-MDR pattern of resistance, as marked by the TB profiler (Supplementary file 3). As expected, *pncA* resistance gene with point mutation *Rv2043c; p.His57Asp* was consistently detected in all *M. bovis* strains due to the established inherent resistance to pyrazinamide. The *rpsA* was another associated gene of resistance to pyrazinamide with point mutation *Rv1630;* p.Ala440Thr detected in all *M. bovis* strains. The highest frequency of resistance genes (in 100% of *M. bovis* strains) was detected for isoniazid, streptomycin, ethambutol and flouroquinolones, followed by delamanid anti-tuberculous drugs (Table 4). The *katG* was the main gene of resistance to isoniazid existing in all *M. bovis* strains, while *ahpC* gene was solely detected in one strain (S20); with illustrated point mutations as revealed in Tables 4 and 3 and Supplementary file 3. The gene of resistance to streptomycin (*rpsl*) exhibited a single point mutation **(**c.-165T > C) that was shared by all *M. bovis* strains. Resistance to ethambutol was represented by four genes (*embB*,* embC*,* embR*,* ubiA)*, that were equally present in *M. bovis* strains, however with distinct mutations as shown in Table 4. Three point mutations in *gyrA (Rv0006)* and one point mutation in *gyrB* (*RV0005;* p.Ala403Ser) constituted the chief determinants of resistance to fluoroquinolones, detected in all strains, while two other point mutations in *gyrB* gene, each was detected in single *M. bovis* strain (p.Val137Ala: S20, p.Trp174: S29). For delamanid, *fbic* was the associated gene of resistance existing in all *M. bovis* strains. Three *fbic Rv1173* point mutations were identified: c.-32 A > G (all strains except S29) and two other mutations (all strains except S5) (Table 3). Four out of five strains (80%) harboured genes of resistance to rifampin. The *M. bovis* strain (S5) harboured both *rpoB* and *rpoC* genes of resistance to rifampin showing point mutations in the form of *Rv0667;* p.Asp435Phe and Rv0668; p.Arg69Pro, respectively. Single detection of each of *rpoB* and *rpoC* gene occured in S28 and in S23, S29 strains, respectively with relevant mutations as demonstrated in Table 3. Two genes of resistance to PASA (*folC* and *ribD*) were detected in single *M. bovis* strain (S5).

**Table 3:** Patterns of anti-tuberculous resistance and conferred resistance genes in *M. bovis* strains (*n* = 5)

M. bovis ID (spoligotype)	M. bovis anti-tuberculous resistance genes (point mutations)									Resistance Pattern
	INH	RF	S	E	PZN^#^	FQ	DE	PASA	C	
S5 BOV_1;BOV_11	*Kat G* (p.Arg463Leu) *ald* (c.-32T > C)	*rpoB* (p.Asp435Phe) *rpoC* (p.Arg69Pro)	*Rpsl* (c.-165T > C)	*embB* (p.Glu378Ala) (p.Asn13Ser) *embC* (p.Thr270Ile) *embR* (c.-207 C > G) *ubiA* (p.Glu149Asp)	*pncA* (p.His57Asp) *rpsA* (p.Ala440Thr)	*gyrA* (p.Glu21Gln) (p.Ser95Thr) (p.Gly668Asp) *gyrB* (p.Ala403Ser)	*fbiC* (c.-32 A > G)	*folC* (p.Ala367Gly) *ribD* (p.Thr146Pro)	*ald* (c.-32T > C)	*Pre-MDR*
S20 BOV_1;BOV_11	*Kat G* (p.Arg463Leu) *ahpC* (p.Pro2Ser) *ald* (c.-32T > C)	-	*Rpsl* (c.-165T > C)	*embB* (p.Glu378Ala) (p.Asn13Ser) *embC* (p.Thr270Ile) *embR* (c.-207 C > G) *ubiA* (p.Glu149Asp)	*pncA* (p.His57Asp) *rpsA* (p.Ala440Thr)	*gyrA* (p.Glu21Gln) (p.Ser95Thr) (p.Gly668Asp) *gyrB* (p.Ala403Ser)	*fbiC* (c.-32 A > G) (g.1305494_1305557del ) (c.2565_2626del)	*-*	*ald* (c.-32T > C) (c.266_266del)	*Pre-MDR*
S23 BOV_1;BOV_11	*Kat G* (p.Arg463Leu) *ald* (c.-32T > C)	*rpoC* (p.Arg69Pro)	*Rpsl* (C-165T > C	*embB* (p.Glu378Ala) (p.Asn13Ser) *embC* (p.Thr270Ile) *embR* (c.-207 C > G) *ubiA* (p.Glu149Asp)	*pncA* (p.His57Asp) *rpsA* (p.Ala440Thr)	*gyrA* (p.Glu21Gln) (p.Ser95Thr) (p.Gly668Asp) *gyrB* (p.Ala403Ser)	*fbiC* (c.-32 A > G) (g.1305494_1305557del ) (c.2565_2626del)	*-*	*ald* (c.-32T > C) (c.266_266del)	*Other*
S28 BOV_1;BOV_11	*Kat G* (p.Arg463Leu) *ald* (c.-32T > C)	*rpoB* (p.Asp435His) *rpoB* (c.1287_1289del)	*Rpsl* (C-165T > C)	*embB* (p.Glu378Ala) (p.Asn13Ser) *embC* (p.Thr270Ile) *embR* (c.-207 C > G) *ubiA* (p.Glu149Asp)	*pncA* (p.His57Asp) *rpsA* (p.Ala440Thr)		*fbiC* (c.-32 A > G) (g.1305494_1305557del ) (c.2565_2626del)	*-*	*ald* (c.-32T > C) (c.266_266del)	*other*
S29 BOV_1;BOV_11	*Kat G* (p.Arg463Leu) *ald* (c.-32T > C)	*rpoC* (p.Arg69Pro)	*Rpsl* (C-165T > C)	*embB* (p.Glu378Ala) (p.Asn13Ser) *embC* (p.Thr270Ile) *embR* (c.-207 C > G) *ubiA* (p.Glu149Asp) *ubiA* (p.Glu149Asp)	*pncA* (p.His57Asp) *rpsA* (p.Ala440Thr)	*gyrA* (p.Glu21Gln) (p.Ser95Thr) (p.Gly668Asp) *gyrB* (p.Ala403Ser) (p.Trp174)	*fbiC* (g.1305494_1305557del (c.2565_2626del)	*-*	*ald* (c.-32T > C) (c.266_266del)	*other*

INH: isoniazid, RF: rifampin, S: streptomycin, E: ethambutol, PZN: pyrazinamide, FQ: fluoroquinolones, DE: delamanid, PASA: para-aminosalycilic acid, C: D-cyclocerine, MDR: multidrug-resistance

**Table 4:** Distribution and frequency of detected anti-tuberculous resistance genes among the *M. bovis* strains

Resistance genes	Anti-TB Drug	Gene locus	Point mutations	WHO confidence Grading of mutation	M. bovis strains	
					Frequency (%) *N* = 5	M. bovis ID
*rpoB*	Rifampin	*Rv0667*	p.Asp435Phe	Group1	1 (20%)	S5
*rpoB*			p.Asp435His	Group2	1 (20%)	S28
*rpoB*			c.1287_1289del in frame_deletion (medium score)	Tier 1	1 (20%)	S28
*rpoC*		*Rv0668*	p.Arg69Pro	Group5	3 (60%)	S5, S23, S29
*KatG*	Isoniazide	*Rv1908c*	p.Arg463Leu	Group5	5 (100%)	S5, S20,S23,S28,S29
*ahpC*		*Rv2428*	p.Pro2Ser	Tier 1	1 (20%)	S20
*ald*	D-cycloserine	*Rv2780*	c.-32T > C	NA	5 (100%)	S5, S20,S23,S28,S29
*ald*			c.266_266del (frameshift) high score	NA	4 (80%)	S20,S23,S28,S29
*rpsl*	Streptomycin	*Rv0682*	c.-165T > C	Group5	5 (100%)	S5, S20,S23,S28,S29
*embB*	Ethambutol	*Rv3795*	p.Glu378Ala	Group5	5 (100%)	S5, S20,S23,S28,S29
*embB*			p.Asn13Ser	Group5	5 (100%)	S5, S20,S23,S28,S29
*embC*		*Rv3793*	p.Thr270Ile	Group5	5 (100%)	S5, S20,S23,S28,S29
*embR*		*Rv1267c*	c.-207 C > G	Tier 2	5 (100%)	S5, S20,S23,S28,S29
*ubiA*		*Rv3806c*	p.Glu149Asp	Group5	5 (100%)	S5, S20,S23,S28,S29
*pncA*	Pyrazinamide#	*Rv2043c*	p.His57Asp	Group1	5 (100%)	S5, S20,S23,S28,S29
*rpsA*		*Rv1630*	p.Ala440Thr	Tier 2	5 (100%)	S5, S20,S23,S28,S29
*gyrA*	Fluoroquinolones	*Rv0006*	p.Glu21Gln	Group5	5 (100%)	S5, S20,S23,S28,S29
*gyrA*			p.Ser95Thr	Group5	5 (100%)	S5, S20,S23,S28,S29
*gyrA*			p.Gly668Asp	Group5	5 (100%)	S5, S20,S23,S28,S29
*gyrB*		*Rv0005*	p.Ala403Ser	Group5	5 (100%)	S5, S20,S23,S28,S29
*gyrB*			p.Val137Ala	Tier1	1 (20%)	S20
*gyrB*			p.Trp174	Tier 1	1 (20%)	S29
*fbiC*	Delamanid	*Rv1173*	c.-32 A > G	Tier 2	4 (80%)	S5, S20,S23,S28
*fbiC*			g.1305494_1305557del )(large deletion) high score	Tier 2	4 (80%)	S20,S23,S28,S29
*fbiC*			c.2565_2626del (stop_lost and frameshift) low score	Tier 2	4 (80%)	S20,S23,S28,S29
*folC*	PASA	*Rv2447c*	p.Ala367Gly	NA	1 (20%)	S5
*ribD*		*Rv2671*	p.Thr146Pro	NA	1 (20%)	S5

Group1: associated with resistance, Group2: associated with resistance-interim, Group3: uncertain significance, Group 4: not associated with resistance-interim Group5: not associated with resistance, #: *M. bovis* confers intrinsic resistance to pyrazinamide. PASA: Para-amino salicylic acid. Tier 1: gene sequences that were considered most likely to contain resistance mutations. Tier 2: candidate genes, considered to have a lower, but still reasonable probability of containing resistance mutations.NA: not applicable

### Virulence genetic determinants of the sequenced *M. bovis*

**Fig. 1:**
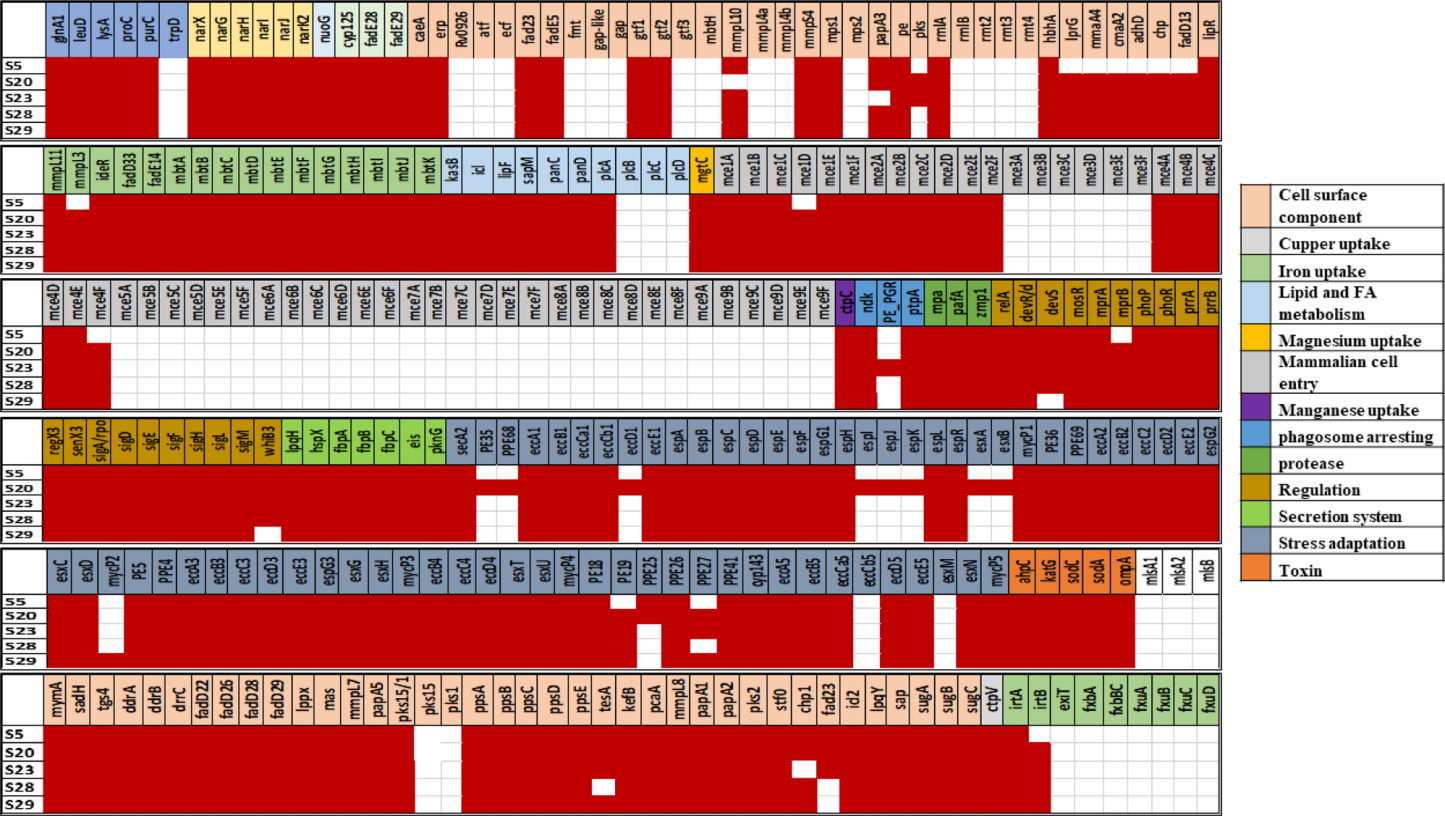
Coloured heat-map demonstrating detailed distribution of virulence genes and assigned virulence classes among the sequenced *M. bovis* strains. The colour code on the side of the figure represents different virulence classes and their correspondent genes. Red shade: present, No shade: absent

Total 85 virulence factors belonging to 18 virulence classes were identified in the searched database (VFBD), as described in Table 5. The major virulence classes were the secretion system, cell surface components, regulation system, mammalian cell entry operons, and stress adaptation. In the five *M. bovis* strains of this study, 75/85 virulence factors assigned to 17 virulence class were represented in 100% of the strains, dominated by the genes of regulatory systems, cell surface components, mammalian cell entry operon and secretory system. These strains completely lacked 10 factors in the form of tryptophan synthesis (aa and purine metabolism), Laminin-binding protein LBP21/Hlp (cell surface component), Exochelin (iron uptake), *mce3-mce5-mce6-mce7-mce8-mce9* (Mammalian cell entry operon) and Mycolactone (toxin) (Table 5). PE family protein (phagosome arresting) was uniquely detected in one strain (S23) (20%). Total annotated virulence genes (*n* = 236) were distributed among whole *M. bovis* genomes, as depicted in Fig. 1 and Supplementary file 4. Whose detailed distribution among the sequenced five *M. bovis* strains is depicted in Fig. 1 and Supplementary file 4. Virulence genes were detected in 100%, 80%, 60%, 40% and 20% of strains at frequencies of 191, 18, 2, 2 and 10, respectively. The highest number of genes was detected in S20 (*n* = 220) and S28 (*n* = 218) strains, followed by S23 (*n* = 211) and S29 strains (*n* = 209). The common virulence genes that existed in 100% of *M. bovis* strains were demonstrated as follows. The highest proportions of virulence genes among the 100% of *M. bovis* strains were found as follows: genes of *secretory system* (*n* = 55/191), *cell surface components* (*n* = 45/191), *regulatory system* (*n* = 17/191) and *Mammalian cell operon* (16/191) in 100% of the *M. bovis* strains (Fig. 1). The highest agreement in distribution of virulence genes (96.5%) was observed between S23 and S28 strains showed, followed by S23 and S29 strains (96.2%).

**Table 5:** Distribution and frequency of virulence factors among sequenced *M. Bovis* (*n* = 5)

Virulence class (Total = 18)	Virulence factors (Total = 85)	F (%)
Amino acid and purine metabolism	Glutamine, leucine, lysine, Proline, purine synthesis	5 (100%)
	tryptophan synthesis	0 (0%)
Anaerobic respiration	Fused nitrate reductase, Nitrate reductase, Nitrate/nitrite transporter	5 (100%)
Anti-apoptosis factor	NuoG	5 (100%)
Catabolism of cholesterol	Cyp125, FadE28, FadE29	5 (100%)
Cell surface components	Carboxylesterase, Exported repetitive protein, GPL locus, Heparin binding hemagglutinin,, Lipoprotein, Methyltransferase, Mycolic acid trans-cyclopropane synthetase, MymA operon, phthiocerol dimycocerosate and PGL phenolic glycolipid biosynthesis and transport, Potassium/proton antiporter, Proximal cyclopropane synthase of alpha mycolates, Sulfolipid-1 biosynthesis and transport, Trehalose-recycling ABC transporter	5 (100%)
	Laminin-binding protein LBP21/Hlp	0 (0%)
Copper uptake	Copper exporter	5 (100%)
Iron uptake	ABC transporter, Heme uptake, Iron-dependent regulator, mycobactin	5 (100%)
	Exochelin	0 (0%)
Lipid and fatty acid metabolism	FASII, Isocitrate lyase, lipase, lipid phosphatase, pantothenate synthesis, phospholipases	5 (100%)
Magnesium uptake	Magnesium transport	5 (100%)
Mammalian cell entry (mce) operons	mce1, mce2, mce4,	5 (100%)
	mc3, mce5, mce6, mce7,mce8, mce9	0 (0%)
Manganese uptake	P(1)-type Mn2 + transporting ATPase	5 (100%)
Phagosome arrest	Nucleoside diphosphate kinase, Tyrosine phosphatase	5 (100%)
	PE family protein	1 (20%)
Protease	Proteasome-associated proteins, Zn + + metallophrotease	5 (100%)
Regulation	DevR/S, MosR, MprA/B, PhoP/R, RegX3, SenX3, sigmaA, sigmaD, sigmaE, sigmaF, sigmaH, sigmaL, sigmaM, WhiB3	5 (100%)
Secreted proteins	19-KD protein, Alpha-crystallin, Antigen 85 complex, Enhanced intracellular survival protein, Protein kinase G	5 (100%)
Secretion system	Accessory secretion factor, ESX-1, ESX-2, ESX-3, ESX-4, ESX-5	5 (100%)
Stress adaption	AhpC, catalase-peroxidase, Cu, Iron-cofactored SOD, Pore-forming protein	5 (100%)
Toxin	Mycolactone	0 (0%)

### Phylogenetic analysis of comparative *M. bovis* genomes

**Fig. 2:**
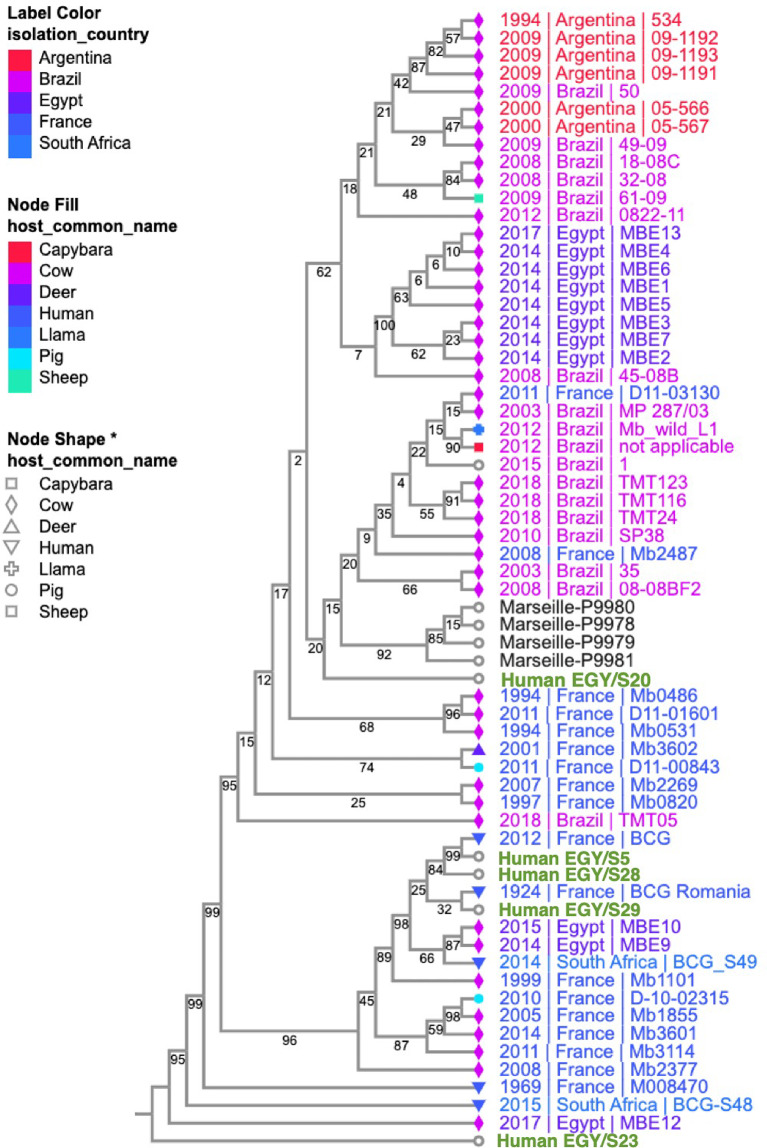
Phylogenetic dendogram of *M. bovis* compared to previously sequenced genomes. The figure demonstrates the genetic likelihood between the five sequenced human *M. bovis* strains from Egypt and a set of 59 previously sequenced *M. bovis* genomes from different hosts and countries. Colour shades and shapes symbolize different *M. bovis* isolation countries and hosts

The details of the phylogeny strains are illustrated in Supplementary file 5, including our sequenced isolates and analyzed dataset of other clinical and veterinary *M. bovis* genomes. The phylogenetic tree shows that three of our *M. bovis* strains (S5, S28, S29) were closely related to some of the previously reported human strains from France, and somewhat related to some cow strains isolated from Egypt. One of our sequenced isolates (S20) was closely related to four human strains isolated from Algeria. Our fifth isolate (S23) was in a separate branch with the closest relatedness to one previously sequenced cow strain from Egypt and another human strain from South Africa. Overall, our *M. bovis* isolates showed diverse phylogeny, but somewhat related to some previously reported either local or regional strains from bovine or human origin (Fig. 2).

## Discussion

*M. bovis* is the chief etiological agent of zoonotic human tuberculosis, which is back on the scene as a growing problem that endangers the health of animals and humans, particularly with rising reports on increasing its virulence and resistance to anti-microbial drugs [24]. Despite of the devastating impact of *M. bovis* on global economy and public health, yet it is not receiving the due attention it should be like *M. tuberculosis*. To date, many developing countries including Egypt suffer a paucity of information about the molecular epidemiology, virulence and resistance of *M. bovis*, particularly the strains isolated from clinical rather than veterinary sources causing zoonotic tuberculosis in human [10].

Out of 112 positive mycobacterial culture samples, five *M. bovis* were isolated with an incidence of 4.46%.This is consistent with prior studies in Egypt reporting bovine human TB at incidence rates of 0.4–6.4% [10, 25]. This aligns with other reports from low-socioeconomic countries in Africa recovering *M. bovis* from 10 to 20% of patients with human TB [5]. Being a zoonotic disease, rates of bovine TB in humans are highly linked to the spread of *M. bovis* among cattles, which can be transmitted to human through aerosol inhalation, ingestion of unpasteurized dairy products or poorly cooked meat. This explains the relatively higher rates of bovine TB in developing countries especially rural areas, where livestock is raised and people have direct and indirect contact with animals [1]. In Egypt, bovine TB in cattle accounted for 0.05% in 1989, which eventually rose to 11–23%, until recently reaching 40.7% comprising a real problem of concern in our country [10, 26]. Likewise, in neighboring North African countries (Algeria and Morocco), livestock infected with *M. bovis* recorded an increase from 3.7% in 2007 to 10.13% in 2021 [27–29]. In our study, we isolated five *M. bovis* strains, all from pulmonary samples, despite the worldwide reports of higher *M. bovis* infection among extra-pulmonary samples, as oral ingestion is the main mode of infection and occurs more often than aerosols inhalation [30–32]. This may explain the high rates of bovine TB reported by a study in Tunisia (76–77%), as it was conducted on extra-pulmonary samples [33], and lower rates (0.6–1.9%) [24] in a study from Ghana among pulmonary samples. As referred to a previous systematic review, zoonotic human TB was reported at a wide from 0.4% [34] to 76.7% [33]. According to this review, variations in reported estimates were owed to the used diagnostic method, where higher rates were more recorded with low discriminatory traditional methods rather than advanced molecular methods [35].

The WGS technology has stratified *MTB* complex into seven lineages assigned as follows: from L1 to L4, L7 (*MTB*), L5 and L6 (*M. africanum*). These lineages may differ in terms of virulence, drug resistance and geographical distribution [36, 37]. Our study identified isolates as *M. tuberculosis var bovis* /spoligotype Bov_1; Bov_11. Before the introduction of WGS to the diagnostic field, *M. bovis* was categorized four regional specific clonal complexes (CC). African 1 and 2 (Af1, Af2: confined to African countries); European 2 (Eu2: found in Iberia) and European 1 (Eu1: widely distributed throughout the world) [36, 38–41]. As proposed by Zimpel et al. (2020), there are four different *M. bovis* lineages (Lb1, Lb2, Lb3, Lb4), which are correspondent to CC Af2, Af1, Eu2 and Eu1, respectively, however they are not entirely represented in these clonal complexes [42]. In agreement with our results, a Brazilian study identified 71 genomes in the database as *M. tuberculosis var Bovis* /spoligotype Bov_1; Bov_11. This could be linked to the importation movement of live cattle from Brazil to Egypt. Of interest, four sequenced genomes in the Brazilian study were identified as BOV AFRI and BOV1 BOV2 [43], although, until recently African strains have rarely been described outside of African countries [42]. Thus, recently observed emergence of African strains in Latin America and Europe necessitates further research to elucidate the origin and transmission dynamics behind the regional introduction of these strains [42]. On the other hand, a previous study in Egypt and another one in Algeria reported the majority of sequenced *M. bovis* isolates as CC Eu2, which originally prevails in Europe and Latin America [10, 27]. Cross-border animal trade and the import of live livestock from Europe to North African countries could provide an explanation. Surprisingly, the two previous studies from Egypt and Algeria lacked the detection of the African strains, which might be due to ill- representation of African isolates in databases of *M. bovis* [10, 27]. The phylogeny of the sequenced five human *M. bovis* genomes in our study was compared with analyzed dataset of other clinical and veterinary *M. bovis* genomes. The phylogenetic analysis revealed a close genetic relatedness of our sequenced human strains to other previously reported cow *M. bovis* strains from Egypt underscoring the zoonotic nature of *M. bovis* and the high potential of transmission from cattle. Furthermore, there was also, close relatedness to some human *M. bovis* strains reported from nearby countries (Algeria and France) denoting regional clustering, which may be related to active people tourism and livestock trade across neighboring countries. Three *M. bovis* strains showed phylogenetic relatedness to some human strains that have been previously reported from France and were recognized as BCG strain, which is known to cause TB in immunocompromised patients. One strain showed a separate cluster that was close to a BCG human strain from South Africa, which may suggest occurrence of a recently evolving strain. It is worth to note that diversification of *M. bovis* lineages is dependent on geographical region rather than the bearing host, which supports the theory of wide host range of *M. bovis* and its capability to infect various mammalian cells [42].

The problem of human tuberculosis is exacerbated by the rising rates of resistance for *MTB*, as well as *M. bovis* to anti-TB drugs, displaying different patterns of resistance ranging from mono-resistance, through multidrug-resistance to extremely-drug resistance [44]. The dilemma of antimicrobial resistance jeopardize all global efforts exerted to manage and control tuberculosis, due to unsuccessful treatment and limited availability of appropriate treatment options [45]. What distinguishes *M. bovis* is its known intrinsic resistance to pyrazinamide, which is a key player in recent regimens of tuberculosis that have managed to reduce the duration of therapy from 1 year to 6 months [44]. In our study, all *M. bovis* strains showed resistance gene mutations to isoniazid, streptomycin, pyrazinamide, ethambutol, fluoroquinolones and cyscloserine, while 80% of strains harbored resistance mutations to rifampin and delamanid. This is concordant to previous studies that reported several resistance gene mutations to first and second anti-TB drugs [11, 46]. The differentiating point from those studies is that we did not encounter gene mutations conferring resistance to aminoglycosides in our study. A prior study in Egypt phenotypically demonstrated resistance to rifampin, isoniazid, streptomycin and ethambutol at rates of 32.8%, 30.8%, 20%% respectively, however, our study was limited by lacked phenotypic susceptibility results that hindered the ability to correlate with the genotypic resistance profile. Nevertheless, the prediction of associated phenotypic resistance can be inferred on genotypic basis, according to the confidence grading of the WHO catalogue [47].

In our study, the mutation detected in the *pncA* gene was the substantial genetic determinant for pyrazinamide resistance to which *M. bovis* is intrinsically resistant, as generally established in the WHO catalogue. Additionally, another point mutation was detected in the *rpsA* gene, whose role in pyrazinamide resistance is debated in literature [48]. As referred to the WHO, *rpsA* gene is one of the genes that comprise low, but justifiable chance of occurring resistance mutations [47]. Isoniazid resistance mutations were detected in *katG* and *ahpC* genes; however with prediction of no association with resistance [47].The uncontrollable and consistent use of isoniazid in treating bovine tuberculosis represent driving factors for occurrence of resistance [43]. For rifampin, confident mutations for resistance were detected in *rpoB* gene, in addition to other less reliable mutations in *rpoB* and *rpoC* genes. As reported in prior studies, all strains showed underlying resistance mutations in *rpsl* gene for streptomycin, and *in embB*,* embC*,* embR* and *ubiA* genes for ethambutol with poor association with resistance according to the WHO [43, 46, 47, 49]. In line with previous reports, we found different *gyrA* and *gyrB* mutations conferring variable levels of associated resistance to quinolones in all *M. bovis* strains [43, 45, 50]. Lately, an upward trend of resistance of *M. bovis* to quinolones has been observed, which might be owed to the overconsumption of quinolones in the treatment of other types of bacterial infections [51]. Interestingly, although delamanid has been recently introduced in therapeutic regimen of MDR-TB, however resistance began to emerge shortly after its use and even before its first debut in the market [43, 52, 53]. In consistence with previous reports, our study detected several mutations in the *fbiC* gene that were designated as candidate genes of low possibility of associated resistance to delamanid, as per the WHO [43, 47, 53]. Notably, although *MTB*C genomes are relatively stable and evolve slowly compared to other microbes [36], yet resistance mutations constantly emerge, which may occur independently of antibiotic exposure [43].

We identified 85 virulence factors belonging to 18 virulence classes using the virulence factor database VFDB online tool. These are associated with important physiological processes such as the secretion system, cell surface components, regulation system, mammalian cell entry operons, and stress adaptation. Our strains showed positivity for most of the virulence factors tested in the VFDB, as 75/85 virulence factors assigned to 17 virulence class were represented in 100% of the strains. These finding explains the virulence and the human pulmonary infection.

As referred by Cheng et al. (2019), high level of virulence factors was directly proportional to the degree of pathogenicity in the mouse model [54]. The proteins expressed by these genes are mainly involved in secretion and cell surface transport, like trehalose-recycling ABC transporter and phthiocerol dimycocerosates (PDIMs) and phenolic glycolipids (PGLs). Trehalose-recycling ABC transporter is responsible for acquisition of host sugars, which is important for M. tuberculosis infection, PDIMs and PGLs are structurally- related complex lipids in the mycobacterial cell wall and present only in pathogenic mycobacteria. Interestingly all three were present in all of our *M. bovis* strains. The *phoPR* system shows differential expression between *M. bovis* and M. tuberculosis. *phoPR* is a master regulator of virulence through regulation of synthesis of many components including the *ESX-1* secretion system and many lipids restricted to pathogenic members of the *MTB*C. The *ESX-1* secretion system exports, among other factors, the dominant T-cell antigens *ESAT-6 (EsxA*) and *CFP-10 (EsxB)* [55]. All of our isolates harbored the *phoP* and *ESX-1* systems and only one isolates harbored additionally the *ESAT-6* and *CFP-10*.

Moreover, Garcia et al. (2021) showed that inactivation of *phoP* in *M. bovis* led to decreased ability of the mutant to block phagosome maturation after engulfment by macrophages and had altered expression of 70 including genes involved in sulfolipid biosynthesis and transport, oxidative stress responses, and *ESX-1* secretion [56]. The *M. bovis phoP* mutant was also more sensitive to acid stress than the wild type, as the *phoP* controls the production and secretion of ammonia that buffers against acid stress, which mimics the in vivo environment.

The effective management and control of human and animal tuberculosis warrants the provision of robust and comprehensive diagnostics. The WGS technology outperforms the standard narrow-scale molecular assays that are designed with limited genetic targets and poor ability of discrimination between closely related strains [36, 57–60]. WGS is a revolutionary technology that achieved tangible milestones in infectious diseases stepping from the field of research towards the applied field of human and animal health [61–63]. The high-resolution power and fine-scale genomic analysis of WGS makes it a superior technology endorsed by the WHO in studying microbial epidemiology, exploring emerging antimicrobial resistance an investigating outbreaks and transmission events [52]. Lately, the WGS has been employed by high-income countries in the control programs of *MTB* and *M. bovis* for more informed decisions and actions [61].

In this work, we tried to benefit the merits of sequencing technology in characterizing the entire genome of human *M. bovis* clinical isolates. This was to highlight the prevalent lineages in our country, find the genetic relatedness to strains from other countries, and explore the underlying genetic drivers of virulence and resistance. However, our study had limitations that included low number of sequenced isolates, probably due to the low isolation rate encountered for this pathogen, especially from human source. Furthermore, we lacked the opportunity of performing phenotypic drug susceptibility testing or targeted validation of identified gene mutations, as the resources demanded for implementing these procedures were not available. Nevertheless, as mentioned before, phenotypic resistance can be deduced on genotypic basis, as referred to the confidence grading of the WHO catalogue. After all, there is a long path to go through further large-scale researches to gain profound understanding of *M. bovis* genomic diversities, dynamics of transmission, pathogenicity and resistance. This shall be in concerted efforts to design new assays that optimize animal screening and active surveillance.

## Conclusion

All sequenced *M. bovis* isolates were of spoligotype BOV_1; BOV_11. All sequenced strains harboured anti-TB drug-resistance gene mutations for isoniazid, streptomycin, ethambutol, fluoroquinolones and cycloserine, while most isolates had gene mutations for rifampin and delamanid. The dominant virulence genes belonged to classes of secretion system, cell surface components and regulation system. The phylogenetic analysis of our sequenced isolates, together with a set of previously sequenced *M. bovis* genomes from different geographical areas revealed genetic relatedness of our isolates to bovine strains from Egypt and human strains from nearby countries in the region, including France, Algeria and South Africa. This finding may suggest potential cross-border transmission or shared ancestor of *M. bovis* strains. Larger-scale studies of *M. bovis* are strongly urged to better understand the epidemiology and transmission dynamics of zoonotic tuberculosis in our geographical region.

## Electronic supplementary material

Below is the link to the electronic supplementary material.

Supplementary Material 1

Supplementary Material 2

Supplementary Material 3

Supplementary Material 4

Supplementary Material 5

## Data Availability

The datasets of the sequenced M. bovis analyzed during this study are available in GenBank under the Bioproject PRJNA1019495. Genome sequences have been deposited in the NCBI with Genomic Accession numbers listed in Table 2 and Supplementary files 1 and 2.

## Abbreviations

MTB: M. tuberculosis
MTBC: M. tuberculosis complex
WGS: Whole-genome sequencing
WHO: World Health Organization
INH: Isoniazid
RF: Rifampin
PZA: Pyrazinamide, S:streptomycin
FQ: Fluoroquinolone
DE: Delamanid
PASA: Para amino- salycilic acid
C: Cyclocerine
VFDB: Virulence factor database
NCBI: National Center for Biotechnology Information
BV-BRC: Bacterial and Viral Bioinformatics Resource Center
MDR: Multidrug-resistant
