# Supplementary material for: Genetic diversities and drug resistance in Mycobacterium bovis isolates from zoonotic tuberculosis using whole genome sequencing
Source: BMC Genomics. 2024 Nov 1;25:1024. doi: 10.1186/s12864-024-10909-8 (PMC11529264; doi:10.1186/s12864-024-10909-8)
Supplement: Supplementary file 1 — Supplementary Material 1 [file 12864_2024_10909_MOESM1_ESM.pdf]

An official website of the United States government
Here's how you know

Submissions > SUB13933478 > Report

Download all files from this report

BioSample Accession	File	Status	Message	Genome Accession
SAMN38024412	28_contigs.fasta	Processing	• PGAP file (28_contigs00000000.bgpipe.output_243294.sqn, 28_contigs00000000.bgpipe.output_243294.gb)	JAXIQB000000000
SAMN38024413	S10_contigs.fasta	Processing	• PGAP file (S10_contigs	JAXIQC000000000

Feedback

			00000000.bgpipe.output_39634.sqn, S10_contigs00000000.bgpipe.output_39634.gb)	
SAMN38024414	S22._contigs.fasta	Processing	• PGAP file (S22__contigs00000000.bgpipe.output_224552.sqn, S22__contigs00000000.bgpipe.output_224552.gb)	JAXIQD000000000
SAMN38024415	S25_contigs.fasta	Processing	• PGAP file (S25_contigs00000000.bgpipe.output_47536.sqn, S25_contigs00000000.bgpipe.output_47536.gb)	JAXIQE000000000
SAMN38024416	S27_contigs.fasta	Processing	• PGAP file (S27_contigs00000000.bgpipe.output_175159.sqn, S27_contigs00000000.bgpipe.out	JAXIQF000000000

			put_175159.gb)	
SAMN38024417	S37._contigs.fasta	Processing	• PGAP file (S37__contigs00000000.bgpipe.output_267760.sqn, S37__contigs00000000.bgpipe.output_267760.gb)	JAXIQG000000000
SAMN38024418	S51._contigs.fasta	Processing	• PGAP file (S51__contigs00000000.bgpipe.output_4958.sqn, S51__contigs00000000.bgpipe.output_4958.gb)	JAXIQH000000000
SAMN38024419	S56._contigs.fasta	Processing	• PGAP file (S56__contigs00000000.bgpipe.output_228306.sqn, S56__contigs00000000.bgpipe.output_228306.gb)	JAXIQI000000000
SAMN38024420	S59._contigs.fasta	Processing	• PGAP file (S59__contig	JAXIQJ000000000

			s00000000.bgpipe.output_62338.sqn, S59__contigs00000000.bgpipe.output_62338.gb)	
SAMN38024421	S63._contigs.fasta	Processing	• PGAP file (S63__contigs00000000.bgpipe.output_203072.sqn, S63__contigs00000000.bgpipe.output_203072.gb)	JAXIQK000000000
SAMN38024422	S66._contigs.fasta	Processing	• PGAP file (S66__contigs00000000.bgpipe.output_27398.sqn, S66__contigs00000000.bgpipe.output_27398.gb)	JAXIQL000000000

FOLLOW NCBI

Connect with NLM

National Library of Medicine
8600 Rockville Pike
Bethesda, MD 20894

Web Policies
FOIA
HHS Vulnerability Disclosure

Help
Accessibility
Careers

NLM NIH HHS USA.gov

Last revision: 1.192.0
