# Supplementary material for: Genetic diversities and drug resistance in Mycobacterium bovis isolates from zoonotic tuberculosis using whole genome sequencing
Source: BMC Genomics. 2024 Nov 1;25:1024. doi: 10.1186/s12864-024-10909-8 (PMC11529264; doi:10.1186/s12864-024-10909-8)
Supplement: Supplementary file 2 — Supplementary Material 2 [file 12864_2024_10909_MOESM2_ESM.pdf]

An official website of the United States government
Here's how you know

Submission Portal

Submissions > SUB13854918 > Report

Download all files from this report +

BioSample Accession	File	Status	Message	Genome Accession
SAMN37519951	S1_contigs.fasta	Processing		JAVTLN000000000
SAMN37519953	S11_contigs.fasta	Processing		JAVTLO000000000
SAMN37519954	S13_contigs.fasta	Processing		JAVTLP0000[illegible]00
SAMN37519955	S14_contigs.fasta	Processing		JAVTLQ0000[illegible]00
SAMN37519956	S15_contigs.fasta	Processing		JAVTLR000000000

Feedback

SAMN37519957	S16_contigs.fasta	Processing		JAVTLS000000000
SAMN37519958	S17._contigs.fasta	Processing		JAVTLT000000000
SAMN37519959	S18_contigs.fasta	Processing		JAVTLU000000000
SAMN37519960	S2_contigs.fasta	Processing		JAVTLV000000000
SAMN37519961	S20_contigs.fasta	Processing		JAVTLW000000000
SAMN37519964	S23._contigs.fasta	Processing		JAVTLX000000000
SAMN37519965	S24._contigs.fasta	Processing		JAVTLY000000000
SAMN37519967	S26_contigs.fasta	Processing		JAVTLZ000000000
SAMN37519969	S29._contigs.fasta	Processing		JAVTMA000000000
SAMN37519970	S3_contigs.fasta	Processing		JAVTMB000000000
SAMN37519971	S30._contigs.fasta	Processing		JAVTMC000000000
SAMN37519972	S31._contigs.fasta	Processing		JAVTMD000000000
SAMN37519973	S32._contigs.fasta	Processing		JAVTME000000000
SAMN37519974	S33._contigs.fasta	Processing		JAVTMF000000000
SAMN37519975	S34_contigs.fasta	Processing		JAVTMG000000000
SAMN37519976	S35._contigs.fasta	Processing		JAVTMH000000000

SAMN37519977	S36._contigs.fasta	Processing		JAVTMI000000000
SAMN37519980	S39_contigs.fasta	Processing		JAVTMJ000000000
SAMN37519981	S4_contigs.fasta	Processing		JAVTMK000000000
SAMN37519982	S40_contigs.fasta	Processing		JAVTML000000000
SAMN37519983	S5_contigs.fasta	Processing		JAVTMM000000000
SAMN37519986	S53._contigs.fasta	Processing		JAVTMN000000000
SAMN37519987	S54._contigs.fasta	Processing		JAVTMO000000000
SAMN37519990	S58_contigs.fasta	Processing		JAVTMP000000000
SAMN37519992	S6_contigs.fasta	Processing	• modified fasta sequences (ForeignContaminationModified_S6_contigs.zip)<br>• fixed foreign contaminations (FixedForeignContaminations_S6_contigs.txt)	JAVTMQ000000000
SAMN37519993	S60._contigs.fasta	Processing	• modified fasta sequences (ForeignContaminatio	JAVTMR000000000

			nModified_S60__contigs.zip)<br>• fixed foreign contaminations (FixedForeignContaminations_S60__contigs.txt)	
SAMN37519994	S61._contigs.fasta	Processing		JAVTMS000000000
SAMN37519995	S62._contigs.fasta	Processing	• modified fasta sequences (ForeignContaminationModified_S62__contigs.zip)<br>• fixed foreign contaminations (FixedForeignContaminations_S62__contigs.txt)	JAVTMT000000000
SAMN37519997	S65_contigs.fasta	Processing		JAVTMU000000000
SAMN37520000	S69_contigs.fasta	Processing		JAVTMV000000000
SAMN37520004	S72_contigs.fasta	Processing		JAVTMW000000000
SAMN37520005	S73_contigs.fasta	Processing		JAVTMX000000000

SAMN37520006	S74._contigs.fasta	Processing		JAVTMY000000000
SAMN37520007	S75._contigs.fasta	Processing		JAVTMZ000000000
SAMN37520008	S76_contigs.fasta	Processing		JAVTNA000000000
SAMN37520009	S77._contigs.fasta	Processing	• modified fasta sequences (ForeignContaminationModified_S77__contigs.zip)<br>• fixed foreign contaminations (FixedForeignContaminations_S77__contigs.txt)	JAVTNB000000000
SAMN37520011	S8_contigs.fasta	Processing		JAVTNC000000000
SAMN37520012	S87._contigs.fasta	Processing	• modified fasta sequences (ForeignContaminationModified_S87__contigs.zip)<br>• fixed foreign contaminations (FixedForeignContaminations_S87__contig	JAVTND000000000

			s.txt)	
SAMN37520013	S9_contigs.fasta	Processing		JAVTNE000000000
SAMN37519950	28_contigs.fasta	Deleted	• Deleted at submitters request, contamination (FixedForeignContaminations_28_contigs.txt, ForeignContaminationModified_28_contigs.zip, Contamination_28_contigs.txt, RemainingContamination_28_contigs.txt)	
SAMN37519952	S10_contigs.fasta	Deleted	• Deleted at submitters request, contamination (Contamination_S10_contigs.txt, RemainingContamination_S10_contigs.txt)	
SAMN37519962	S21._contigs.fasta	Deleted	• remaining contaminations (Remaini	

			ngContamination_S21__contigs.txt)<br>• genome size errors (GenomeSize_S21__contigs.txt)<br>• contaminations (Contamination_S21__contigs.txt)<br>• modified fasta sequences (ForeignContaminationModified_S21__contigs.zip)<br>• fixed foreign contaminations (FixedForeignContaminations_S21__contigs.txt)<br>• has errors	
SAMN37519963	S22._contigs.fasta	Deleted	• Deleted at submitters request, contamination (FixedForeignContaminations_S22__co	

			ntigs.txt, ForeignContaminationModified_S22__contigs.zip, Contamination_S22__contigs.txt, RemainingContamination_S22__contigs.txt)
SAMN37519966	S25_contigs.fasta	Deleted	• Deleted at submitters request, contamination (Contamination_S25_contigs.txt, RemainingContamination_S25_contigs.txt)
SAMN37519968	S27_contigs.fasta	Deleted	• Deleted at submitters request, contamination (Contamination_S27_contigs.txt, RemainingContamination_S27_contigs.txt)
SAMN37519978	S37._contigs.fasta	Deleted	• Deleted at submitters re

			quest, contamination (Contamination_S37__contigs.txt, RemainingContamination_S37__contigs.txt)
SAMN37519979	S38_contigs.fasta	Deleted	• remaining contaminations (RemainingContamination_S38_contigs.txt)<br>• genome size errors (GenomeSize_S38_contigs.txt)<br>• contaminations (Contamination_S38_contigs.txt)<br>• modified fasta sequences (ForeignContaminationModified_S38_contigs.zip)<br>• fixed foreign contaminations (FixedForeignConta

			minations_S38_contigs.txt)<br>• has errors
SAMN37519984	S51._contigs.fasta	Deleted	• Deleted at submitters request, contamination (Contamination_S51__contigs.txt, RemainingContamination_S51__contigs.txt)
SAMN37519985	S52_contigs.fasta	Deleted	• genome size errors (GenomeSize_S52_contigs.txt)<br>• has errors
SAMN37519988	S56._contigs.fasta	Deleted	• Deleted at submitters request, contamination (Contamination_S56__contigs.txt, RemainingContamination_S56__contigs.txt)
SAMN37519989	S57_contigs.fasta	Deleted	• genome size errors (Ge

			nomeSize_S57_contigs.txt)<br>• has errors
SAMN37519991	S59._contigs.fasta	Deleted	• Deleted at submitters request, contamination (FixedForeignContaminations_S59__contigs.txt, ForeignContaminationModified_S59__contigs.zip, Contamination_S59__contigs.txt, RemainingContamination_S59__contigs.txt)
SAMN37519996	S63._contigs.fasta	Deleted	• Deleted at submitters request, contamination (FixedForeignContaminations_S63__contigs.txt, ForeignContaminationModified_S63__contigs.zip, Contaminatio

			n_S63__contigs.txt, RemainingContamination_S63__contigs.txt)
SAMN37519998	S66._contigs.fasta	Deleted	• Deleted at submitters request, contamination (Contamination_S66__contigs.txt, RemainingContamination_S66__contigs.txt)
SAMN37519999	S68_contigs.fasta	Deleted	• genome size errors (GenomeSize_S68_contigs.txt)<br>• modified fasta sequences (ForeignContaminationModified_S68_contigs.zip)<br>• fixed foreign contaminations (FixedForeignContaminations_S68_contigs.t

			• xt) • has errors	
SAMN37520001	S7_contigs.fasta	Deleted	• genome size errors (GenomeSize_S7_contigs.txt) • has errors	
SAMN37520002	S70._contigs.fasta	Deleted	• genome size errors (GenomeSize_S70__contigs.txt) • has errors	
SAMN37520003	S71._contigs.fasta	Deleted	• genome size errors (GenomeSize_S71__contigs.txt) • has errors	
SAMN37520010	S79_contigs.fasta	Deleted	• genome size errors (GenomeSize_S79_contigs.txt) • has errors	
SAMN37520014	S91._contigs.fasta	Deleted	• genome size errors (GenomeSize_S91__contigs.txt) • has errors	

FOLLOW NCBI

Connect with NLM

National Library of Medicine
8600 Rockville Pike
Bethesda, MD 20894

Web Policies
FOIA
HHS Vulnerability Disclosure

Help
Accessibility
Careers

NLM NIH HHS USA.gov

Last revision: 1.192.0
